# “The familiar taste of poison”: a qualitative study of multi-level motivations for stimulant use in sexual minority men living in South Florida

**DOI:** 10.1186/s12954-023-00787-w

**Published:** 2023-04-26

**Authors:** Leah Davis-Ewart, Ji-Young Lee, Michael Viamonte, Josè Colon-Burgos, Audrey Harkness, Mariano Kanamori, Dustin T. Duncan, Susanne Doblecki-Lewis, Adam W. Carrico, Christian Grov

**Affiliations:** 1 Miller School of Medicine, University of Miami, Miami, FL USA; 2 Department of Mental Health Law and Policy, University of South Florida, Tampa, FL USA; 3 Robert Stempel School of Public Health and Social Work, Florida International University, Miami, FL USA; 4 School of Nursing and Health Studies, University of Miami, Miami, FL USA; 5 Mailman School of Public Health, Columbia University, New York, NY USA; 6 Graduate School of Public Health and Health Policy, City University of New York, 55 West 125th Street, Office 812, New York, NY 10027 USA

**Keywords:** HIV, Stimulant use, Sexual minority men, Stigma, Qualitative research

## Abstract

**Background:**

In the US, stimulant use is associated with a 3–6 times greater rate of HIV seroconversion in sexual minority men (SMM) than in those who do not use stimulants. Annually, 1 in 3 SMM who HIV seroconvert will be persistent methamphetamine (meth) users. The primary objective of this qualitative study was to explore experiences of stimulant use in SMM living in South Florida, a high priority region for the Ending the HIV Epidemic initiative.

**Methods:**

The sample included 25 SMM who use stimulants, recruited via targeted ads on social networking apps. Participants completed one-on-one semi-structured qualitative interviews, conducted from July 2019 through February 2020. A general inductive approach was used to identify themes relating to experiences, motivations, and overall relationship with stimulant use.

**Results:**

Mean age of participants was 38.8, ranging from 20 to 61 years old. Participants were 44% White, 36% Latino, 16% Black and 4% Asian. Most participants were born in the US, self-identified as gay, and preferred meth as their stimulant of choice. Themes included: (1) stimulants as cognitive enhancements for focus or task completion, including transitioning to meth after first using prescription psychostimulants; (2) unique South Florida environment where participants could be open regarding their sexual minority status while also being influential on their stimulant use; (3) stimulant use as both stigmatizing and a coping mechanism for stigma. Participants anticipated stigma by family and potential sexual partners due to their stimulant use. They also reported using stimulants to cope with feelings of stigma due to their minoritized identities.

**Conclusion:**

This study is among the first to characterize motivations for stimulant use in SMM living in South Florida. Results highlight both the risk and protective factors of the South Florida environment, psychostimulant misuse as a risk for meth initiation, and the role of anticipated stigma on stimulant use in SMM. Understanding stimulant use motivations can help to shape intervention development. This includes developing interventions that address individual, interpersonal, and cultural factors that drive stimulant use and increase risk of HIV acquisition.

*Trial registration* NCT04205487.

## Background

Stimulant use (referred to here as usage of methamphetamine, crack-cocaine and/or powder cocaine) among sexual minority men who have sex with men (SMM) is up to 20 times more prevalent than in the general population [1]. Stimulants, especially methamphetamine (meth), can be used by SMM for increased energy and for sexual pleasure, reducing inhibitions, prolonging sexual encounters and has been associated with HIV-vulnerability through related risk behaviors [2, 3]. Miami-Dade, Broward and Palm Beach counties (South Florida) ranked first in US HIV incidence from 2015 to 2019, is consistently in the top five metropolitan statistical areas in the US [4] and is a priority jurisdiction in the Ending the HIV Epidemic (EHE) plan [5, 6]. So far, the dominant focus of prior research examining stimulant use in SMM has focused on sexualized drug use. However, stimulant use motivations are often more contextualized and nuanced than sexual enhancement motivations. To address HIV-related disparities in SMM in South Florida, interventions should consider stimulant use behaviors. Developing a more comprehensive, multi-level understanding of individual, interpersonal and cultural determinants of SMM’s stimulant use could guide efforts to optimize the benefits of HIV pre-exposure prophylaxis (PrEP) and decrease HIV incidence in high priority areas.

Previous findings have highlighted the role of stimulants in sexualized drug use for SMM, such as “party and play (PnP)” or “parTy” in North America [3, 7, 8]. Combining stimulant such as meth with amyl nitrites (i.e., poppers) and erectile dysfunction drugs (PnP) can be used to increase libido, social connectedness and enhance sexual experiences[3, 7, 8]. As such, stimulant use is also associated with increased HIV-related risk behaviors, including condomless anal sex, multiple sexual partners, and sex with partners of an unknown HIV serostatus [1, 2]. Thus, stimulant use poses a risk for HIV transmission and is associated with a 3 to 6 times greater rate of HIV seroconversion than in SMM who do not use stimulants [9–14]. In one US-based prospective study, one in three SMM who HIV seroconverted in the first year of follow-up reported meth use [2].

Prominent theories such as the Cognitive Escape Model propose that SMM may be psychologically motivated to escape or avoid chronic stressors such as HIV and minority stressors with substance use [8, 15]. SMM may encounter stressors such as discrimination, internalized stigma, and victimization because of their minoritized status in a heteronormative society [3, 16, 17]. Stimulants and other substance use may be viewed from the lens of self-medicating, cognitive disengagement and/or as a coping mechanism for negative experiences and negative moods [18, 19]. Research also finds that stimulant use and minority stress processes may impede utilization of HIV prevention services including PrEP uptake or adherence [8, 20]. Understanding the role stimulant use plays in stress and coping may be important for addressing both mental health and HIV-related disparities in SMM.

Additionally, we suggest that affiliation with gay culture may have important risk and protective implications for stimulant use in SMM. Previous studies have examined the perceptions and motivations surrounding stimulant use in SMM. In 2005, Kurtz found that meth use in SMM living in Miami was associated with the desires to decrease loneliness and feel more physically attractive in the face of aging and illness [21]. Parsons and colleagues found that initiation into meth use was often for social purposes, not sexual, and that many first-time users had limited knowledge of the meth and often compared it to cocaine [22]. Cocaine usage was also associated with meth initiation, as some participants in Kelly and colleagues’ research described using meth due to diminishing effects of cocaine [23]. Finally, Stanton and colleagues found that stimulant use may be described from an interpersonal lens, with use for intimacy and relationship building, empowerment, identity affirmation and a sense of community belonging [3].

With the persistence of stimulant use among SMM [8, 24, 25] and high HIV incidence and prevalence in South Florida, it is important move beyond the narrative of sexualized drug use as the only driver of meth and other stimulant use. A local, current, contextual understanding of multi-level factors contributing to substance use is needed to understand how stigma, environment and culture impact SMM who use stimulants. This includes examining the experiences of substance use initiation and the role of South Florida as a risk environment. Insight into the context in which SMM use stimulants, particularly meth, can help guide interventions that address the environments in which stimulants are used and help decrease it’s the negative impact of stimulants on HIV-related outcomes including PrEP uptake, adherence, and HIV incidence. Therefore, the objective of this qualitative study was to explore experiences of SMM with stimulant use initiation, motivations for stimulant use, and consequences of stimulant use for SMM living in South Florida.

## Methods

This study took place within the broader context of the formative phases of a randomized control trial of PrEP Readiness Interventions for Supporting Motivation (PRISM), an intervention to increase PrEP uptake and decrease stimulant use among SMM in South Florida The goal of this formative work was to assess the acceptability and feasibility of a motivational interviewing and contingency management intervention components of the intervention, including questions regarding PrEP use and perceptions, substance use motivations and initiation experiences. As such, the researchers conducted semi-structured interviews to learn about participants’ lived experiences with stimulant use, for those who both do and do not use PrEP. During enrollment, and before randomization into the trial, participants completed a one-on-one, in-person semi-structured qualitative interview about 60 min in length, conducted from July 2019 through February 2020.

Participants were identified via an adapted, targeted sampling strategy [26], where the researchers recruited through social networking apps geolocated to Miami-Dade and Broward Counties, flyers at the local needle exchange center, and from the consent-to-contact databases of other University of Miami (UM) HIV prevention programs. Cisgender men, at least 18 years old, who met the CDC guidance for PrEP eligibility [27] (at least one male condomless anal sex partner in the last 6 months who was not their primary partner), who reported using any amount of meth, crack-cocaine or cocaine at least once in the last 3 months, and who reported being HIV-negative were included in the study. Those who reported living with HIV, and/or who had sex with only a primary partner were excluded from the study. Participants who were eligible were contacted by research staff to participate in a baseline assessment that included the in-depth interview described in this manuscript. Participants completed an informed consent prior to data collection, and they were compensated $40 for their time. The Institutional Review Board at UM approved all study procedures.

Participants were given a supplementary quantitative survey to derive additional information, including demographics, Alcohol, Smoking and Substance Involvement Screening Test (ASSIST) scores [28], and the PrEP continuum [29]. The interviews followed a semi-structured interview guide assessing the acceptability and feasibility of the contingency management intervention components, the overall goal of the formative research. For the purpose of the current study, we are focusing our analysis on additional questions in the interviews about participants’ history of substance use (including their stimulant use initiation), their preferred type of stimulant used, and their likes and dislikes about the stimulants they reported preferring. All interviews were audio recorded with participants’ consent, and transcribed verbatim by a certified transcription company. A member of the research then verified transcripts against the original recording for accuracy.

The research team of six was diverse with respect to race/ethnicity, sexual orientation, training levels and discipline. Team members held sexual, gender and racial/ethnic minority affirming views. The senior authors provided ongoing training in qualitative methods, study objectives and SMM health disparities. Interviewers were trained in qualitative methods and supervised by the study’s senior authors.

The general inductive approach was used to identify themes relating stimulant use and drug initiation experiences [30]. Codes were derived inductively, starting with a thorough reading of the transcripts and identification of relevant information expressed by the participants. An initial codebook was developed by the primary data analyst after a comprehensive reading of all transcripts. Next, a random sample of five interviews was selected for a second data analyst to code for inter-rater agreement. The two researchers reviewed and discussed any discrepancies in the coding, emergent themes, and the need for refined definitions. The researchers accepted all codes where there was agreement and came to a consensus for areas of non-agreement based on their discussions; they revised the codebook, and re-coded the transcripts based on the revised codebook. A second random sample of five interviews was then selected to determine inter-rater agreement. The analysts reached an inter-rater agreement of 93% in the second round of coding, and transcript codes were finalized [31]. Agreement was defined as whether identical codes were applied to the selected text by both coders. Once inter-rater agreement was reached, all 25 interviews were coded by either one of the 2 analysts based on the final codebook, and saturation was deemed to have been reached using established guidelines for determining coding saturation [32].

## Results

Participants included 25 SMM who use stimulants. The average age of participants was 38.8 and ranged from 20 to 61 years old. A little more than half of participants were persons of color (Latino (36%), Black (16%) and Asian (4%)) and 44% were White. Most participants were currently using a daily, oral PrEP (64%), while 24% had never used PrEP and 12% had discontinued their PrEP regimen. Based on the ASSIST, 44% of participants scored a moderate risk severity for meth use, 24% scored moderate risk severity for cocaine/crack-cocaine, and 44% scored moderate risk severity for inhalants. Most participants were born in the US, described themselves as gay, were uninsured and were employed full-time (Table 1).

**Table 1:** Demographic characteristics of participants (N = 25)

	*Mean*	SD
Age	38.76	13.8

	*n*	%
Race/ethnicity		
Asian	1	4
Black	4	16
White	11	44
Latino	9	36
Country of origin		
US born	16	64
Foreign born	9	36
Sexual orientation		
Gay	20	80
Bisexual	5	20
Stimulant of choice		
Meth	17	68
Crack-cocaine	2	8
Cocaine	6	24
ASSIST scores-moderate risk severity		
Meth	11	44
Coke/crack	6	24
Inhalants	11	44
PrEP status		
Current	16	64
Former	3	12
Never	6	24
Employment status		
Full time	9	36
Part time	2	8
Student	6	24
Unemployed-other	8	32
Insurance status		
Insured	11	44
Uninsured	14	56

There were several notable themes from participants. While stimulant use for sexual enhancement remained a motivation for stimulant use, participants expressed other reasons and drivers behind their use. Participants talked about their stimulants in the context of cognitive enhancements for focus or task completion, including transitioning to meth after first using prescription psychostimulants. They described South Florida as being a unique environment where participants could be open regarding their sexual minority status while also being influential on their stimulant use. They also talked about stimulant use as both stigmatizing and a coping mechanism for stigma. Specifically, participants anticipated stigma by family and potential sexual partners due to their stimulant use. They also reported using stimulants to cope with feelings of stigma due to their minoritized identities.

### Initiation experiences and transitioning to stimulants for cognitive enhancements

This theme refers to the ways in which SMM first initiated their substance use. A little over half of participants reported that cocaine was their first stimulant used. For instance, one participant stated, “Okay, first one was coke, and then after that was crack, and then meth is the last drug I'm addicted of, the trail or you want to say, whatever you wanna call it” (White, 22 years old, current PrEP and meth use).

Alternatively, some participants discussed starting to use stimulants as cognitive enhancements for focus or task completion (Table 2). For example, “I have been diagnosed, late diagnosis, with ADD. And so before that, I had used [meth] for self-medication, for productivity and stuff like that…and I never use [meth] for recreational purposes… I would use it to perform my jobs. It just helps me concentrate and I just feel more able to finish my jobs, or whatever I'm doing,” (White, 50 years old, current PrEP and meth use).

**Table 2:** Multi-level factors impacting stimulant use

Level of influence	Theme	Illustrative quote
*Individual*	Stimulant use as a cognitive enhancement	“I was still being prescribed Adderall, so I didn’t feel the need to even want to try it [meth] again…Then when they did take me off of the Adderall, I went back [to using meth]—those 9 months, I was like, ‘God, there’s got to be something else I can do in the meantime.” And then I remembered my friend. I was like, ‘Hey, you think you can like [connect me with some meth]?’ And then that’s when I went and tried it again—this time smoking it—and it was a lot less of a shock to the system.” (Latino, 34 years old, no PrEP use, meth use)
	Stimulant use as coping mechanism for minority stress	“It eases my depression. You know, to me, it makes me feel like I'm a better person.” (Black, 49 years old, no PrEP use, coke use)
*Interpersonal*	Stimulant use during sexual encounters Not a primary theme but noted in “Discussion” section	“The guys I end up fucking would usually have it [meth]. So, I'd say, "What’s up? Can I hit that?" And they'd say, "Sure, whatever." I mean, is it—I think using meth just pertains to, like, the sexual activities, sexual activities in general here in South Florida. I feel like everyone that's gay and—I mean, everyone that's gay in South Florida is on [social networking app] and they all do meth.” (Latino, 24 years old, current PrEP and meth use)
	Stimulant use as coping mechanism for minority stress • Perceived stigma by friends and family	“But in the gay community, I feel like [there is a lot of stigma]. Most of the people that I see around, it's [meth] very much related to depression and being lonely. And being sad for not being able to have a family, or thinking about it. Or being, like, judged by society and other people, or maybe their family members, or their friends… But I see lots of people are very depressed, and that's why they do so much drugs.” (White, 27 years old, former PrEP and current meth use)
	Stimulant use as coping mechanism for minority stress • Anticipated stigma from potential sexual partners	“[There is the] stigma of it, or judgment of people, every time that you think about bringing it up, you don't know if that's gonna be something that, like, hangs up, or blocks you, or whatever, so- I think I kind of consciously and subconsciously screen who I meet based on [whether] I think that they do [meth] or not… because I'll probably want to do it at some point while I'm with them, so… even if they aren't… it's probably better [to know now] if they aren't just gonna get up and walk out, (laughs).” (White, 35 years old, current PrEP and meth use)
*Cultural Environment*	Culture of Stimulant Use in South Florida’s Gay Community • South Florida as LGBTQ + affirming	“[I came here] to be able to be myself. To be able to be gay in peace… There's a little bit of danger for people being gay in Brazil. You know, other people won't like it, and then they will do crimes against you, or hurt you, or anything like that. Here it's more safe and open. I never felt any prejudice [here].” (White, 27 years old, former PrEP, meth use)
	Culture of Stimulant Use in South Florida’s Gay Community • Perceived ubiquity of stimulant use in South Florida SMM community	“It's everywhere [in South Florida]. Every gay house, every gay community. Gay roommates living together, they all, they all have PrEP. And they- they do [meth] for having sex and all that.” (White, 27 years old, former PrEP, meth use)

Most participants who reported using stimulants for cognitive enhancement indicated that they transitioned to meth after first using prescription stimulant medications commonly prescribed for attention deficit hyperactivity disorder (ADHD) (e.g., Adderall). For example:

*I'm already like familiar with Adderall, from when I was growing, taking it like when as a teenager. So, um, mostly it's like if I need to get something done or like, um, if I need to like paint all night or clean,* (Latino, 21 years old, no PrEP use, meth use).

*... back in 2009 or 2010, but, like, I had an Adderall prescription then, so I just did it [meth] once in a while, and... that was it, but then, last spring, the doctor that was prescribing me 120 milligrams of Adderall a day retired (laughs) and nobody else was prescribing it, and I came down here, and slept, like, the first week I was here and still wasn't waking up, and then someone I met on [a social networking app] had it [meth] with him. I woke up, and I was like, ‘Hey, this might be not a bad way [to make up for the Adderall], so-* (White, 34 years old, current PrEP use, meth use).

*I was still being prescribed Adderall, so I didn’t feel the need to even want to try it [meth] again…Then when they did take me off of the Adderall, I went back [to using meth]—those 9 months, I was like, ‘God, there’s got to be something else I can do in the meantime.” And then I remembered my friend. I was like, ‘Hey, you think you can like [connect me with some meth]?’ And then that’s when I went and tried it again—this time smoking it—and it was a lot less of a shock to the system.* (Latino, 34 years old, never on PrEP, meth use).

### Culture of stimulant use in South Florida’s gay community

Participants commented on how living in South Florida allowed them both to be more open about their sexual minority status compared to their home countries or other states, while also being influential on their stimulant use. For SMM who immigrated or migrated to Miami from less open or affirming countries or states, participants described South as a place where sexual minorities can live in peace. “[I came here] to be able to be myself. To be able to be gay in peace… There's a little bit of danger for people being gay in Brazil. You know, other people won't like it, and then they will do crimes against you, or hurt you, or anything like that. Here it's more safe and open. I never felt any prejudice [here]” (White, 27 years old, former PrEP, meth use). Another participant said, “I am French. I moved here Miami almost four years ago… Um, I basically started my gay life here in Miami. Not that much in France. And then I discovered my gay life here in Miami. Um, and so far, I'm super happy here,” (White, 24 years old, never on PrEP, cocaine use).

However, participants also connected stimulant use to perceived social acceptance and perceived prevalence specifically within the South Florida gay community. Participants described stimulant use as a regular part of the night life and social life for SMM in South Florida.

*Everybody's on something and they have their [drug of] choice. So it's either you're doing GHB while you're having sex and doing Tina, and hanging out with the muscle boys of South Beach, or you're doing cocaine, or you're, you know, drinking at the same time. But you're on something and you're part of the crowd and it's up to you how far you want to take it. Then you have the South Beach queens and in Fort Lauderdale, Wilton Manors is full of Tina. It's the bears, the muscle queens, and they see it as part of a sex drug. So. to them, crystal meth or Tina or whatever they want to call it, either they slam* [inject] *it, they smoke it, they put it up their ass* [‘booty bump’]*, they do whatever* (37 years old, Latino, current PrEP use, cocaine use).

*It's everywhere [in South Florida]. Every gay house, every gay community. Gay roommates living together, they all, they all have PrEP. And they- they do [meth] for having sex and all that,* (White, 27 years old, former PrEP use, meth use).

Another participant expressed how meth use was common during sexual encounters and hard to turn down when frequently presented:

*The guys I end up fucking would usually have it [meth]. So, I'd say, "What’s up? Can I hit that?" And they'd say, "Sure, whatever." I mean, is it—I think using meth just pertains to, like, the sexual activities, sexual activities in general here in South Florida. I feel like everyone that's gay and—I mean, everyone that's gay in South Florida is on [social networking app] and they all do meth* (Latino, 24 years old, current PrEP and meth use).

### Stimulant use as stigmatizing or coping mechanism for stigma

Participants reported that their stimulant use could be thought of as both stigmatizing and as a coping mechanism for stigma. First, participants discussed their fear of family finding out about their stimulant use. For instance, one participant reported, “It's really bad. If my parents, if they know this, they will kill me, like… So when you think about the moral thing, you know, your family and everything, I think it's really bad,” (Asian, 26 years old, current PrEP and meth use). Others described anticipating stigma or rejection from potential partners regarding stimulant use during sexual situations. Multiple participants noted that as they talked to potential partners online, they were careful about how they responded to questions about “partying,” due to concerns that people would screen them out based on their answers to questions.

*Some guys I'll talk to and the first thing they ask is ‘Do you party?’ I'm pretty open about whether I want to party so I'll either say yes or no… But if they use the capital T and write ‘parTy,’ then you know that they're using meth. But if they don't, then you don't know if they're using coke or if they aren't using anything… So when they use [the capital T in party] I automatically know that it's meth. When they don't, that's when I'm a little more guarded about whether I am going to tell them [that I party]—especially if they don't use it [meth] and I still want to have sex with them* (White, 21 years old, current PrEP and meth use).

*[There is the] stigma of it, or judgment of people, every time that you think about bringing it up, you don't know if that's gonna be something that, like, hangs up, or blocks you, or whatever, so- I think I kind of consciously and subconsciously screen who I meet based on [whether] I think that they do [meth] or not… because I'll probably want to do it at some point while I'm with them, so… even if they aren't... it's probably better [to know now] if they aren't just gonna get up and walk out, (laughs)* (White, 35 years old, current PrEP and meth use).

Others described substance use as a coping mechanism for experiences of stigma related to their sexual identity or minority stress:

*But in the gay community, I feel like [there is a lot of stigma]. Most of the people that I see around, it's [meth] very much related to depression and being lonely. And being sad for not being able to have a family, or thinking about it. Or being, like, judged by society and other people, or maybe their family members, or their friends… But I see lots of people are very depressed, and that's why they do so much drugs*, (White, 27 years old, former PrEP use, meth use).

*It eases my depression. You know, to me, it makes me feel like I'm a better person* (Black, 49 years old, never on PrEP, cocaine use).

### Love-hate relationship with stimulants

Participants were asked to describe their overall relationship with their stimulant of choice, considering the likes and dislikes discussed in the interview. Most participants labeled their relationship with stimulants as “love-hate.” They discussed their overarching knowledge that stimulant use was bad for their health in the long run, but their current desire for the pleasure associated with stimulant use counterbalanced the negative impact. As one participant stated, his relationship with meth was:

[It’s a] *Familiar taste of poison. Exactly what it would be like. It's, you want to do it, like you have to do it, like you see you love it, but at the same time, for as much as you love it and you dance with it, it kills you. Like you know slowly but surely like it's, it's killing you-* (White, 33 years old, current PrEP and meth use).

Another participant described his relationship with meth as follows:

*Oh, she's [meth] a pretty one… But you have to watch your back.* (Latino, 58 years old, current PrEP and meth use).

## Discussion

This qualitative study examined the complex cultural, interpersonal and individual level determinants of stimulant use among SMM living in South Florida. Although sexual enhancement remained a primary motivation for stimulant use [33–35], the perceived ubiquity of stimulants in the South Florida SMM community potentiates exposure risk for use of stimulants, particularly in sexualized contexts. Narratives also revealed those with a history of prescription psychostimulants for symptoms of ADHD described it as increasing risk for meth and other stimulant use. Finally, some participants indicated that they commonly navigated stigma related to their stimulant use within the gay community and reported using stimulants to manage sexual minority stress experiences.

Use of meth for ADHD self-medication was a new and important finding from our results. Participants framed their meth use as a form of self-medication, either as an amphetamine/dextroamphetamine substitute or use for focus and energy. Meth can be used to self-treat ADHD symptoms [36], as prescription stimulants and meth have similar chemical compounds and impact on biological functioning [37]. ADHD can increase vulnerability to future substance use, including meth, due to self-medication and behavioral disinhibitions [37, 38]. Previous research shows that people who use meth are 2–6 times more likely to have ADHD than non-users [36, 39], 47% of people who use meth report previous prescription stimulant misuse [40], and that SMM who misuse prescription stimulants without co-use of meth have a 2.5 greater odds of meth use at 12 months [37]. Participants in the current study indicated they used meth to get out of bed, complete errands and assignments, or for focusing on tasks. However, they also used meth and other stimulants for sexual enhancement. The co-occurring usage of meth for both sex and task-oriented behaviors noted in our results is a novel contribution to the literature and merits further study.

Our results also highlight the role of South Florida as a potential risk environment for stimulant use among SMM. As Miami is an EHE priority jurisdiction, there is a renewed need for multi-level interventions targeting risk contexts in South Florida such as social norms surrounding stimulant use, use in social spaces and pressure on social networking applications where exposure risk is high for stimulant use. Finally, addressing stigma as a motivation and consequence of stimulant use is crucial. Although SMM feel they can be open about their sexual minority status in Miami, they still have residual feelings of stigmatization from their family and former communities that can lead to stimulant use as a coping mechanism. They also face rejection from their peers and potential sexual partners because of said usage, thereby posing a double bind. Previous research has also linked intersectional stigma regarding stimulant use, perceived judgement, and social structural barriers as negatively impacting both PrEP uptake, adherence and persistence [8, 41, 42]. One strategy for alleviating stigma could be linkage to harm reduction programs where men can safely disclose stimulant use without fear of social rejection, as well as counseling for stigmatizing feelings due to marginalization as a sexual minority.

Participants in this study often described the role of living and participating in the South Florida gay community as facilitators to their stimulant use. Given Miami’s designation as a EHE priority region, understanding the impact of South Florida as an environmental risk factor for substance use and HIV is important. South Florida is a tri-county area composing on Miami-Dade, Palm Beach and Broward Counties. This sprawling urban area offers availability to South Beach in Miami and Wilton Manors in Broward, both established resort communities with a large population of sexual minorities, high prevalence of substance use, and high HIV incidence [43–45]. Previous research shows that South Florida gay communities may offer both risk and protective factors to their residents: while SMM neighborhoods may have higher rates of meth use and condomless anal sex they may also provide protection against substance use disorders, potentially because residents who can afford living in SMM friendly South Florida communities tend to be White and more affluent than those living in the surrounding urban areas [46]. This concept of the risk and protection provided by South Florida was reiterated by our results, as many participants believed that their engagement in the community was both positive for their identity and negative on their substance use. Participants were able to openly expresses their sexuality in South Florida without fear of discrimination, and yet they also felt like it regularly brought them into contact with stimulants and made it hard to abstain from using. Although previous work has also documented the prevalence of stimulant use in South Florida, in particular linking meth to the party subculture in and around the Miami area [21, 47], to our knowledge there is no other current research looking at the context of South Florida as a specific factor in stimulant use and HIV in SMM.

Participants also acknowledged stimulant use as both a cause and effect of stigma. Participants used stimulants as a coping mechanism for internalized, anticipated and enacted experiences of stigma due to their minority status. This finding supports the research surrounding substance use as function of self-medication for minority stressors or as a form of cognitive escape [3, 8, 15, 17]. Prior studies with HIV-positive SMM have shown that feelings of guilt and shame have a bidirectional relationship with stimulant use: higher levels of shame are associated with a slower decrease in stimulant use over time, and higher levels of guilt are positively associated with increased stimulant use [48]. Participants in the current study may be using stimulants as a way to deal with negative self-perceptions, feelings of internalized homophobia, or judgement and rejection from family and friends [49, 50]. Participants also noted that they were concerned about the reaction of potential sexual partners, friends, and family members once their stimulant use was known. Previous qualitative studies have noted how SMM may screen potential sexual partners online to filter out those considered heavy drug users or riskier sexual partners [51]. Research with SMM in Miami also noted the loss of long-term relationships and friendships with continued meth use [21]. Participants in the current study anticipated this interpersonal stigma, and selectively concealed or disclosed their stimulant use accordingly. Research shows that intersectional stigma related to race, sexual minority status and substance use are negatively associated with HIV vulnerability, including prevention, care and treatment outcomes [41]. Addressing the impact of stigma will be important for optimizing substance use and PrEP uptake interventions. For those using stimulants as a coping mechanism, treatment options may focus on managing minority stress processes to address negative reinforcement of stimulant use. For those experiencing anticipated stigma and rejection, treatment options may involve assisting men who use stimulants with disclosing use to prospective partners in a way that decreases experiences of rejection. As an extension, addressing feelings of enacted, internalized and anticipated stigma can help increase the ability to discuss PrEP with a medical providers, and thereby PrEP uptake and adherence [41, 52, 53]. Additionally, interventions should help SMM seek supportive relationships outside of sexualized context where they can be open with other gay men about their meth use, including through harm reduction groups.

Understanding SMM’s motivations for stimulant use is important for developing interventions to address stimulant motivations that may increase sexual risk taking, decrease PrEP uptake, adherence and persistence, and make SMM more likely to acquire HIV. Future initiatives should focus on addressing the individual, interpersonal and cultural norms in South Florida that can facilitate stimulant use, thus intervening on the negative effects of stimulants on HIV-related outcomes such as PrEP uptake and adherence. First, as younger, especially racial/ethnic minority, SMM experience high rates of HIV diagnoses combined with lower PrEP uptake and persistence [54–56], the association between PrEP, meth and psychostimulant misuse should be further explored. Next, mixed-methods research should be used to further our understanding of the role internalized and anticipated stigma play in concealment of substance use and other behaviors that impact HIV vulnerability. Quantitative research should include objective indicators for substance use, including biomarkers, to mitigate the discrepancy between qualitative narratives of severe substance use and quantitative results indicating little to moderate risk. Additionally, future initiatives may include understanding ways to reduce exposure risk on social networking apps and other spaces where men meet romantic and sexual partners. Finally, increasing enrollment of Black and Asian participants in future studies will help better understand contextual differences in stigmatizing experiences and substance use motivations that may impact PrEP-related disparities in uptake and adherence that are exacerbated by substance use.

Our findings should be understood in light of their limitations. First, although the study is focused on stimulant use overall, many of participants primarily used meth. Crack and cocaine may have different short-term and long-term effects when compared to meth, and people who use crack or cocaine may not have the same motivations for use or lived experiences as those who use meth. Next, we enrolled participants who self-reported their HIV status. Verification with an HIV test would have improved validity but was out of the scope of this formative portion of the research study. Additionally, we excluded participants who reported only having sex with one main partner. We recognize that HIV transmission within main partnerships is also possible. Furthermore, while we have a diverse group of men in both age and race/ethnicity, our findings from men living in South Florida may not be generalizable to other areas that are more rural, less diverse or more affluent. Generalizability is generally not a goal of qualitative research [31]. Finally, other forms of HIV prevention beyond PrEP, such as treatment as prevention (TasP), should be explored to create a status neutral approach to ending the HIV epidemic [57].

## Conclusions

Overall, our results help elucidate motivations for, and experiences with stimulant use among a population of SMM living in South Florida, a US HIV epicenter. First, our results help move the literature beyond the dominant narrative of sexualized drug use as the only driver of meth and other stimulant use. Second, it highlights the risk and protective factors associated with the gay community. Third, we have a novel focus on stigma as a chronic stressor that men experience related to their stimulant use as well as something they are motivated to escape via stimulant use. Understanding stimulant use motivations can help to shape intervention development, and in particular, developing interventions that address individual (e.g., task focusing), interpersonal (e.g., coping with stigma, sexual enhancement), and cultural (e.g., the cultural context of South Florida where stimulant is common among SMM) factors that drive stimulant use. To our knowledge, this study is the first to examine stimulant use among SMM as an outcome of stigma, cognitive enhancements and as an interaction within the context of urban South Florida.

## Data Availability

The datasets used and/or analyzed during the current study are available from the corresponding author on reasonable request.

## Abbreviations

ADHD: Attention deficit hyperactivity disorder
ASSIST: Alcohol, Smoking and Substance Involvement Screening Test
EHE: Ending the HIV Epidemic Plan
HIV: Human immunodeficiency virus
PrEP: Pre-exposure prophylaxis
SMM: Sexual minority men
UM: University of Miami
